# Wearable Sensors to Evaluate Autonomic Response to Olfactory Stimulation: The Influence of Short, Intensive Sensory Training

**DOI:** 10.3390/bios13040478

**Published:** 2023-04-16

**Authors:** Lucia Billeci, Chiara Sanmartin, Alessandro Tonacci, Isabella Taglieri, Lorenzo Bachi, Giuseppe Ferroni, Gian Paolo Braceschi, Luigi Odello, Francesca Venturi

**Affiliations:** 1 Institute of Clinical Physiology, National Research Council of Italy (IFC-CNR), Via Moruzzi 1, 56124 Pisa, Italy; 2 Department of Agriculture, Food and Environment, University of Pisa, Via del Borghetto 80, 56124 Pisa, Italy; 3 Interdepartmental Research Centre “Nutraceuticals and Food for Health”, University of Pisa, Via del Borghetto 80, 56124 Pisa, Italy; 4 Institute of Life Sciences, Sant’Anna School of Advanced Studies, 56127 Pisa, Italy; 5 Good Senses s.r.l., Galleria V. Veneto, 9, 25128 Brescia, Italy; 6 Centro Studi Assaggiatori Società Cooperativa, Galleria V. Veneto, 9, 25128 Brescia, Italy; 7 Interdepartmental Centre for Complex Systems Studies, University of Pisa, Largo Bruno Pontecorvo, 2, 56126 Pisa, Italy

**Keywords:** autonomic nervous system, electrocardiogram, galvanic skin response, smell, sensory analysis, tasting panel, wearable sensors

## Abstract

In the last few decades, while the sensory evaluation of edible products has been leveraged to make strategic decisions about many domains, the traditional descriptive analysis performed by a skilled sensory panel has been seen to be too complex and time-consuming for the industry needs, making it largely unsustainable in most cases. In this context, the study of the effectiveness of different methods for sensory training on panel performances represents a new trend in research activity. With this purpose, wearable sensors are applied to study physiological signals (ECG and skin conductance) concerned with the emotions in a cohort of volunteers undergoing a short, two-day (16 h) sensory training period related to wine tasting. The results were compared with a previous study based on a conventional three-month (65 h) period of sensory training. According to what was previously reported for long panel training, it was seen that even short, intensive sensory training modulated the ANS activity toward a less sympathetically mediated response as soon as odorous compounds become familiar. A large-scale application of shorter formative courses in this domain appears possible without reducing the effectiveness of the training, thus leading to money saving for academia and scientific societies, and challenging dropout rates that might affect longer courses.

## 1. Introduction

Currently, the global market is highly competitive in any sector concerned with our lives, including the food industry—where sensory evaluation of edible products is gaining importance—as it is leveraged to make strategic decisions about many domains. These decisions include quality control, the possibility to undergo improvements in the product, to design new items, and to eventually change or re-engineer production processes. All this has been rapidly changing with respect to the past, as it involves the consumer more than ever, taken as a reference in terms of their acceptance of the products, and setting up the benchmark for industrial performance against competitors [1,2,3,4,5].

Traditionally, the decisions taken by consumers rely on the information projected from the environment into their human body. In such a framework, food scientists might not consider the influence of thinking processes on sensory assessment, mainly focusing on stimulus-dependent techniques (the so-called “bottom-up approach”), including scaling or triangle tests for evaluating smell, taste, and flavor [6,7]. Overall, this approach is mainly used when attempting to define the main sensory features of an edible compound for a precise group (e.g., a cohort characterized by a given culture or age). In this regard, sensory analysis is framed in the evaluation of the organoleptic properties of a substance, namely its “attributes” (ISO 5492, 2008), so that for many decades the descriptive analysis of a food, performed with trained evaluators (COI/T.20/Doc. No 4/Rev.1, 2007), has been the gold standard for sensory assessment [8,9].

In this framework, such panelists should be capable of providing a description of the food with training, which is deemed to suppress the individual perception contributing to the information provided, therefore guaranteeing a description of the direct sensory effects brought about by the food investigated [10].

A skilled sensory panel is therefore created in several steps, including recruitment, selection, training and qualification, which is performed on a pool of assessors, possibly not requiring particularly specific conditions (ISO 8586, 2014). This selection of sensory assessors can take up to 120 h of intense testing, depending on the testing demanded and the product complexity [2,8]. In addition, intensively trained assessors are often able to describe the products in a different manner with respect to the usual consumers, as they rely on different, often peculiar sensory features, and are able to detect very subtle differences between two or more samples [5].

Due to such reasons, descriptive analysis was seen to be too complex and unaffordable in terms of time and costs for the industry, making it largely unsustainable in most cases [2,8,11,12]. To cope with this—and in particular to provide a practical, feasible, and affordable response to the requirements and needs of food technology industry in this specific domain—in the last few decades, faster, more affordable methodologies than descriptive analysis have been developed, including the flash profile (FP) method, Napping^®^, sorting, projective mapping, intensity scales, and open-ended questions [5]. Beyond this, more recently, a research line has started to rise, aimed at studying the effectiveness of sensory training on panel performances at different degrees [1,6,13,14].

With respect to the other human senses, olfaction is able to fully label the environment with positive or negative attributes, similar to what occurs with emotions, with which it shares important anatomic links and relationships [15,16,17,18,19,20]. As such, to this extent, the study of the physiological response to olfactory stimuli is key. A number of strategies can be adopted, with relevant advantages and associated drawbacks [21,22,23].

The investigation on physiological reactions could be performed by focusing on biomedical signals, which are extracted using wearable sensors; this, in turn, being an acceptable, affordable, and reliable alternative to most grounded methods. Such signals include, albeit not being limited to, electrocardiogram (ECG) and galvanic skin response (GSR), which non-invasively allow the study of autonomic nervous system (ANS) activity [24,25].

In this context, in a previous study [20], we verified the effectiveness of wearable sensors to measure the impact of a traditional 65 h panel training performed in a three-month period on physiological responses (ECG and GSR) during olfactory assessment performed by judges before and after training.

**Figure 1:**
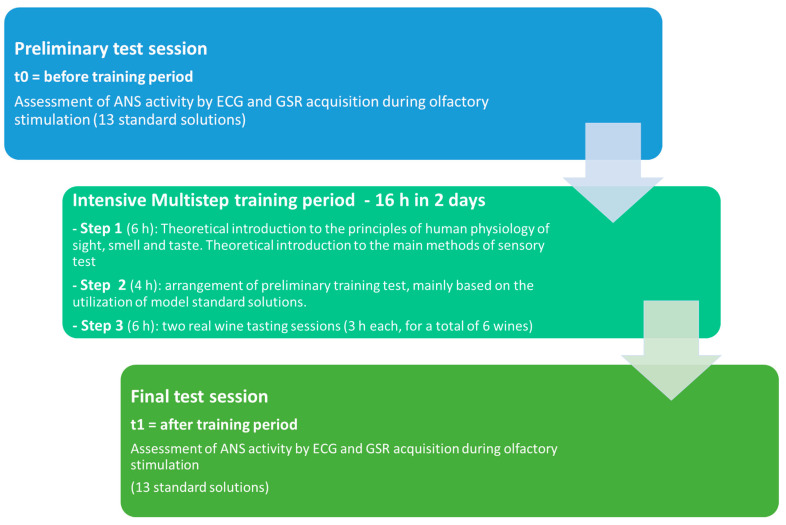
Overall experimental design.

Starting from our previous experimental evidence, the aim of this research was to verify the impact of an intensive 16 h panel training completed in two consecutive days, as is described in Figure 1. According to the protocol previously proposed [20], wearable sensors were used to study physiological signals during olfactory assessment in a cohort of volunteers before and after the sensory training. The results were finally compared with that which was previously reported [20] to verify if the short training can represent a more convenient and time-saving, but reliable, alternative for industrial applications in panel training.

The methodology of this study is outlined in Section 2 (Figure 1), with the experimental results displayed in Section 3. Related discussion is presented in Section 4, and the paper summarizes with its conclusions in Section 5.

## 2. Materials and Methods

### 2.1. Subjects

Seventeen subjects were initially recruited. Two of them did not perform the test session at the final assessment due to personal reasons. Four additional subjects were excluded due to poor ECG signal quality. Thus, the final sample consisted of 11 subjects (7 females, 4 males, age range: 25–63).

### 2.2. Training Description

Selection and training of the panelists involved in the present study was performed according to the Good Senses commercial procedure, which is based on a normalized technical procedure that is specific for qualified judge training, as is reported in the literature, with a few amendments [26].

As such, an intensive two-day multi-step training period was set up, aiming at selecting a group of participants characterized by a level of motivation high enough during the whole activity (level required: attendance at 100% of training sessions), together with a minimum level of sensory skills, which are required for the activities of wine tasting and related sensory descriptions (including visual, aroma, and taste attributes).

To the aims of the present work, the intensive training of the 17 participants enrolled was arranged as follows, over two consecutive days: (i) Step 1 (6 h): The methodologies mainly utilized for mostly diffused sensory tests were introduced and discussed together with a description of the main fundamentals of the human physiology of senses (i.e., sight, smell, and taste). (ii) Step 2 (4 h): The olfactory ability of each panelist (i.e., detection thresholds (sensory acuity); scoring consistency; verbalization skills; odor and flavor memory) were checked by the application of a model standard solution. (iii) Step 3 (6 h): Two wine tasting sessions were performed in a quiet room, properly ventilated. During both sessions, three different wines, both white and red ones, were tested by panelists using a specific sensory sheet, as previously described [27]. Moreover, the panelists described, in detail, even the olfactory profile of each tasted wine in a free manner, in order to make themselves familiar with the main commonly employed wine descriptors [28]. The overall experimental design, including the sensory assessment, is displayed in Figure 1.

### 2.3. Solutions Used for the Olfactory Stimulation

To induce olfactory stimulation, during activities T0 and T1, ten different model solutions (Table 1) were provided to volunteers according to a previously reported protocol [20]. During the activities, each judge was asked to smell each solution for a fixed time and to identify the smell character of each solution when possible.

According to the aim of the research activity, the abovementioned model solutions (Table 1) were prepared exactly as reported in our previous research publication about the role played by a long multi-step training activity on the ANS [20].

As previously reported, widespread raw materials, easily available at the supermarket (i.e., flower distilled essences, different fruit juices, fresh vegetables, and fresh and dried fruits) were added to a neutral commercial white wine base. In our experience, this approach allows the reproduction of a more real tasting experience than that which is eventually obtained by using model solutions that are produced starting from chemically pure standards diluted in artificial model wine (Table 1).

### 2.4. Procedure for ANS Assessment

The activation of the ANS in response to olfactory stimulation was assessed by acquiring two physiological signals using wearable devices: i) the electrocardiogram (ECG), which relies on the electrical activity of the heart, and ii) the galvanic skin response (GSR), which is related to the activation of the sweat glands and, in turn, leads to some electrical activity of the skin.

The procedure for ANS assessment was already published elsewhere by the same research group [20]. Specifically, two testing sessions were performed, respectively, before (T0) and after the training period (T1).

Each session included the following three phases:

(i) Baseline (3′ duration): At the beginning of the recording, the subjects sat on a chair in a comfortable way and were asked to stay still and relaxed;
(ii) Task (6′ 40″ duration): During the administration of the compounds, 10 model solutions (previously described) were given to the panelists for the detection of odors. Each of the proposed solutions was administered to the individuals, tested for 10” in both nostrils, at an inter-stimulus interval of 30″. This pause time was intended to clean the nasal cavity from the residuals of the previous compound [29]. At the same time, the panelists were asked to report, on a paper sheet, the identifier for each of the compounds presented;
(iii) Recovery (3′ duration): This phase was analogous to the baseline, after task completion.

#### 2.4.1. ECG Acquisition and Processing

**Figure 2:**
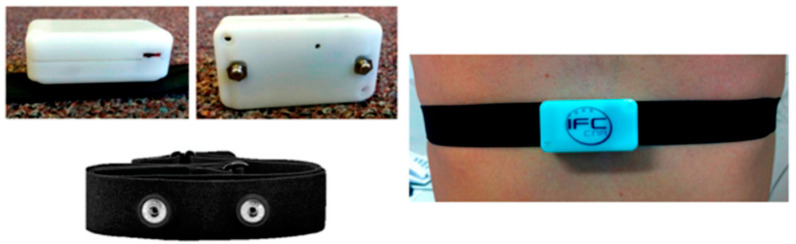
Wearable sensor for electrocardiographic (ECG) signal acquisition based on ECG SHIMMER^TM^ device.

The ECG signal was captured by means of a wearable sensor developed by our team at CNR based on the ECG2 SHIMMER^TM^–based platform (Shimmer Sensing, Dublin, Republic of Ireland). It was interfaced to a commercial fitness-like chest strap (Polar Electro Oy, Kempele, Finland) and programmed to capture the signal according to a sample frequency of 500 Hz. The tool used is displayed in Figure 2 (adapted from [30]).

**Figure 3:**
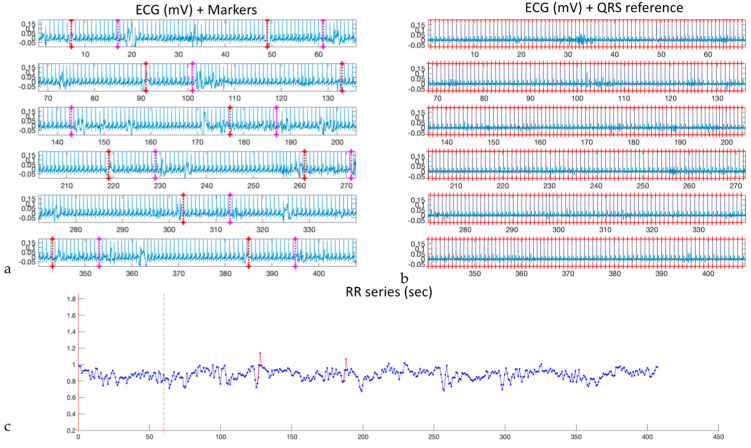
ECG signal processing for a sample signal. (**a**) ECG signal during the task with markers for the beginning (red) and the end (magenta) of olfactory stimulation; (**b**) ECG signal during the task with markers (red) indicating the QRS detection; and (**c**) Reconstructed RR series from the ECG signal (red) and RR series after correction (blue).

The ECG signal was then analyzed using an ad hoc interface in Matlab (The MathWorks, Inc., Natick, MA, USA) (Figure 3). As such, the ECG signals were pre-processed for artifact removal; then, QRS complexes were detected and, finally, the RR series were reconstructed and corrected to remove abnormal, non-sinusoidal beats [31]. From the corrected RR series, different features were extracted, being those most commonly used in the related domain [32,33]. As such, based on the results of our previous study [20], we computed the most significant features in determining the changes in ANS activation. Specifically, the following were taken into account:

- Features on the time domain:
  - Heart rate (HR): number of heart pounds within a time unit. This is measured in beats per minute (bpm), and deals with the sympathetic activity of the ANS [32];
  - Root mean square of the successive differences (RMSSD): measured in ms. This is for the root mean square of the differences between the R-R intervals close to each other. Overall, it matches the parasympathetic branch of the ANS [32];
  - Number of normal R-R intervals differing for more than 50 ms (NN50): this is the number (or the percentage) of the normal R-R intervals of the ECG signal differing for more than 50 ms from each other. Under resting-state short-term recordings, it deals with the parasympathetic activity of the ANS [32];
  - Cardiac sympathetic index (CSI): computed as SD2/SD1. This refers to the sympathetic activity of the ANS. SD1 is the standard deviation of the projection of the Poincaré plot on the perpendicular line to the identity, whereas SD2 is the standard deviation of the projection of the Poincaré plot on the parallel line to the identity [33];
  - Cardiac vagal index (CVI): this is obtained by the Poincaré plot; it is calculated as log10 (SD1*SD2), and refers to the parasympathetic activity of the ANS [33].
- Features on the frequency domain:
  - Low frequency (LF): when taking into account the frequency spectrum of the ECG signal, the LF represents the power spectral density of the ECG signal at low frequencies (0.04–0.15 Hz). According to the literature, it reflects both the activity of both the sympathetic and parasympathetic nervous systems of the ANS [32];
  - High frequency (HF): this represents the power spectral density of the ECG signal at high frequencies (0.15–0.4 Hz), which reflects the parasympathetic activity of the ANS [32];
  - Low-to-high frequency components ratio (LF/HF): this mainly reflects the overall sympathovagal balance of the ANS, although the results need to be framed and justified according to the specific measurement condition [32].

#### 2.4.2. GSR Acquisition and Processing

**Figure 4:**
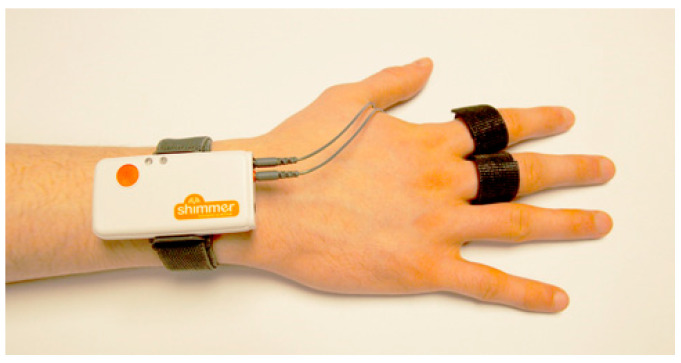
Wearable SHIMMER^TM^ sensor for GSR signal acquisition.

The GSR signal was captured, at 51.2 Hz, by a commercial wearable sensor, the Shimmer3GSR (Shimmer Sensing, Dublin, Republic of Ireland), communicating via Bluetooth to a Windows- or Android-running user interface. To properly acquire the GSR signal, two sensors were positioned on two adjacent fingers of the individual’s non-dominant hand at the phalanx through two soft rings (Figure 4).

**Figure 5:**
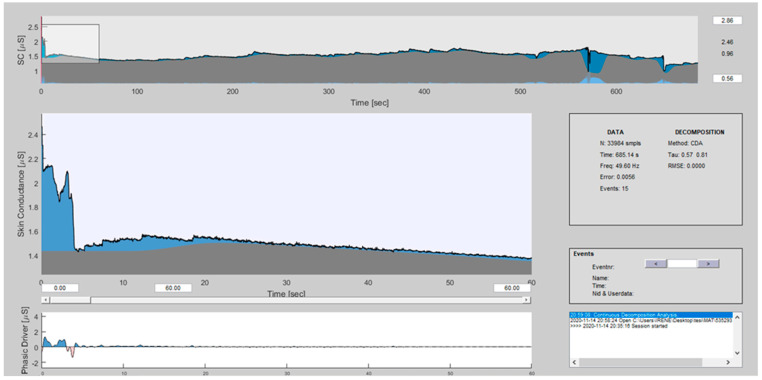
Ledalab interface for GSR signal processing.

Once captured, the GSR signal was analyzed through Ledalab, a Matlab-based tool, which processed this signal [34]. The GSR signal was first filtered with a first-order Butterworth low-pass filter at 5 Hz to remove the high-frequency noise. Then, a continuous decomposition analysis was applied to extract the main components of the GSR signal (Figure 5), namely:

- Global GSR signal: this is the sum of the tonic and phasic components of the GSR signal, measured in microsiemens (µS);
- Tonic GSR: this is mainly related to the slow changes in the electrical skin signal, it is dominant at rest and during most relaxing activities;
- Phasic GSR component: this refers to the quick responses to specific stimuli. In the present study, it can refer to the specific response to sensory (olfactory) stimuli. It is often termed as the skin conductance response (SCR).

### 2.5. Statistical Analysis

The abovementioned features were calculated for the baseline and the recovery condition for the whole acquisition duration. For the task phase, the features were computed for each odor stimulation phase (i.e., “10”) and then the average over the 10 stimuli was applied. Mean values were compared between T0 and T1 to assess the effect of the short sensory training on the activation of the ANS.

Data normality was assessed using the Shapiro–Wilk Test for each of the parameters studied [35]. Since the distribution of data deviated from normality, the Wilcoxon signed-rank test was applied for the comparison between stages, whereas the correlations were calculated through Kendall’s test [36].

For all the analyses conducted, statistical significance was set at *p* < 0.05.

## 3. Results

### 3.1. Odor Identification

The odor identification ability of the subjects enrolled for the present study improved from an average (out of 10) of 4.25 ± 2.24 at T0 to 5.31 ± 2.47 at T1 (*p* = 0.004), highlighting a significant effectiveness of the olfactory training on the identification skills (and on the compounds’ knowledge) of the panelists.

### 3.2. ECG Signal

As mentioned before, the ECG features were extracted and analyzed in terms of differences between T0 and T1 during the three phases of the testing session: Baseline, Task, and Recovery.

The results are reported in Table 2. Most of the features changed from T0 to T1, in particular during Baseline and Task. Furthermore, HR, CSI, and LF/HF decreased at T1 with respect to T0 in such phases, while RMSSD, NN50, and HF increased. These results indicates an overall sympathetic activity decrease and parasympathetic activity increase between the two testing sessions.

**Figure 6:**
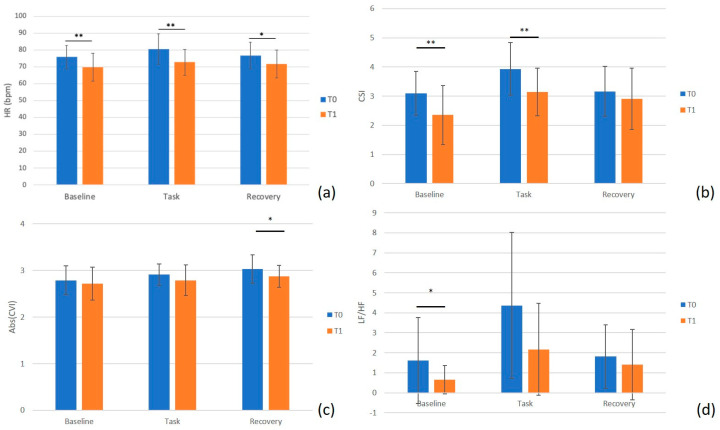
ECG results at T0 and T1 for (**a**) HR, (**b**) CSI, (**c**) CVI, and (**d**) LF/HF features (* *p* < 0.05 and ** *p* < 0.01).

To better elucidate the trends of the features, the most relevant ones are represented in Figure 6.

In addition, we tested for differences between the different phases within a single session (e.g., Baseline vs. Task, Task vs. Recovery), discovering a significant increase for HR at T0 between Baseline and Task (*p* = 0.001), followed by a decrease at Recovery (*p* = 0.003). The same trend was not noticed at T1, where HR increased at Task more slightly than in the previous session (*p* = 0.032), with a non-significant decrease at Recovery (*p* = 0.083). Conversely, the RMSSD showed a marked decrease at T0 during the Task with respect to the basal result (*p* = 0.003), without differences between Task and Recovery (*p* = 0.831). In the case of the RMSSD, T1 displayed the same variations as T0, with Task displaying a lower RMSSD than Baseline (*p* = 0.003), with no differences at Recovery (*p* = 0.465). No significant variations were noticed for the other ECG features.

### 3.3. GSR Signal

As for the ECG signal, the GSR features were also compared between T0 and T1. The results are reported in Table 3. For all the three phases, both the global and tonic GSR decreased from T0 to T1, while the phasic phase did not change significantly.

A comparison was also performed for the GSR features between the different phases within a single testing session. Both global and tonic GSR increased at Task with respect to Baseline at both T0 (*p* < 0.001 for both) and T1 (*p* = 0.005 for the two features). Interestingly, the phasic GSR, matching the stimulus-specific response of the sympathetic branch of the ANS, had a different trend at T0 than at T1. Indeed, at T0, an increase at Task (*p* = 0.010) was followed by a marked decrease at Recovery (*p* = 0.003), whereas at T1 no variations were noticed between Baseline and Task (*p* = 0.569) nor between Task and Recovery (*p* = 0.756), suggesting a different stimulus-specific behavior after the sensory training period.

## 4. Discussion

The present study extends prior knowledge about the effects of olfactory training on ANS responses, a well-known surrogate for emotional assessment in healthy human beings [20]. In fact, thanks to the large-scale availability of easy-to-use tools—which are both pervasive and affordable for the end users—used by either academia or the research institutions in charge of the study of physiological reactions, the application of wearable sensors is continuously growing in this specific domain, providing useful insights on one’s health and physiological reactions even in naturalistic or semi-structured settings [37]. Indeed, such a paradigm would allow the detection of stress exposure and related responses with a minimal annoyance over the probands and, at the same time, enabling data collection from a large number of individuals than that which could be obtained using the gold standard, more sophisticated methods.

In more depth, this approach is deemed particularly useful in the food and drink industry, where the inter-connection between panel questionnaires, chemical sensors, and derived devices (e.g., e-Nose, e-Tongue, e-Eye)—with the study of emotions of panelists and consumers at large—is becoming key, as has been suggested [6]. At the same time, the interaction usually performed by a panelist or a consumer at large with a given edible compound (either food or beverage) should not be significantly altered by the application of a monitoring tool or by the use of gold standard, complex, wired instrumentation that is poorly feasible in the specific framework. This makes the approach described in the present investigation highly applicable and, to the best of our knowledge, ideal in such a use-case scenario, such as the panel testing or the consumer-based neuromarketing, at large.

The present investigation confirms, in a small cohort of individuals undergoing a short, intensive sensory training on wine tasting, the trends previously seen in longer periods of time [20], which have already demonstrated a tendency toward somewhat higher parasympathetic activation during the second session after training with respect to what noticed at the first test, i.e., before the training period. However, the previous paper [20] took into account longer periods of time and less intensive training protocols, different from that which was conceived for the present work. In fact, even after a few days of training, the volunteers enrolled for the current study displayed enhanced identification ability toward odorous compounds dealing with wine-related characteristics, suggesting enhanced familiarity toward such substances. The intervention modulates both familiarity and the response of the ANS. In general terms, in the second assessment, the volunteers displayed more pronounced relaxation toward the trial, as demonstrated by the significant sympathetic withdrawal and vagal activation at T1 than at T0. This is in line with our previous research [20], but could reflect a plethora of psychophysiological phenomena and emotional engagement, which might not be strictly related to the sensory task per se, but could be due to a different approach to the testing session overall. What is particularly important regarding the attitude toward odorous compounds is the comparison between phases at T0 with respect to T1. In this analysis, it is evident from some of the features extracted that, at T1, a tendency toward a lower sympathetic response to odors is present. This is particularly present when we take into account punctual features, which are stimulus-specific, such as the phasic response of the GSR signal, which displayed a completely different behavior during the second testing session with respect to the previous one.

Overall, in both sessions, the task requested smelling an odor and attempting at identifying or characterizing it. This approach featured a request for a special emotional and cognitive engagement, which led to increases in the sympathetic nervous system activation and to some parasympathetic withdrawal occurring within the task. This is coherent not just with the previous experience [20], but also with a significant amount of the literature in this domain [24,38]. It is postulated that such an effect is mainly seen in negative-valence odor-related reactions, where the sympathetic branch of the ANS was seen to increase in its activity [39].

However, the relationship between sympathetic activation and the pleasantness of a given compound is particularly interesting, which is why we have already discussed the close, well-known correlation between familiarity and the pleasantness of olfactory stimuli. Such a correlation was already postulated some decades ago by eminent scientists in the field [40,41,42]; however, this left room for further speculation in terms of the consequentiality between the two dimensions. In this regard, our previous study shed a light on this, contributing the hypothesis for an increased pleasantness, which is matched by the increasingly positive physiological and emotional reactions as much as the target odor becomes familiar. However, the previous paper published involved a study that lasted 3 months, with regular, albeit not intensive, training sessions taking place throughout this period. However, this relatively long time window could have allowed for a small amount of olfactory structures to regenerate, possibly affecting the quality of the results obtained [43,44,45]. In addition, the applicability of such a training strategy, for example, on a cohort of diseased individuals (e.g., those with eating disorders) are potentially disruptive in terms of changing some specific features of the clinical condition. This becomes questionable as time needed to train such individuals over a different approach toward odor and related food compounds rises. Last, but not least, the societies in charge for sensory training often pay high costs to undergo their academic activities, making these poorly affordable for small centers if the timeframe becomes too long. Therefore, the usefulness of shorter, more intensive training sessions can be manifold, and their application can be spread in various domains of sensory sciences, as well as beyond them.

By basically matching the results obtained in the longer training course [20] with the sympathetic hyper activation at T0, followed by a trend toward re-balance between sympathetic and vagal responses at T1, in light of the results reported above about the stimulus-specific response to odors, the present investigation made clear that the effects brought by sensory training on a small cohort of panelists were independently the same from the modality of its fruition. To the best of our knowledge, this is the first ever attempt at proving this in a cohort of panelists engaged into wine tasting, and could serve as a future reference for several domains involving sensory (olfactory) training. Indeed, albeit being considered in light of the study limitations (e.g., the small sample size, the suboptimal technical functioning of the sensors used, the lack of comparison between the effects generated by the single odorous compounds), such advancement of state-of-the-art practices could enable academia and scientific societies to promote short-term sensory training courses, possibly with the same benefits as traditional courses in terms of the knowledge generated, as well as from an emotional point of view in the attitude toward wines in this case, or food if such findings could be confirmed in other matrices. In addition, such retrievals would open the door for the adoption of short sensory training in other domains. For example, if the effect is confirmed also on clinical populations, its usage can serve to create a powerful food literacy in neurological and neuropsychiatric conditions, where the adoption of correct lifestyles and approaches toward food is key to maintain, or restore, an acceptable level concerning the quality of life. Those include, albeit not being limited to, dementia [46], neurodegenerative disorders [47,48], neurodevelopmental disorders such as autism [49], and, last but not least, eating disorders [50].

One of the strengths of our study is represented by the application of wearable devices. Wearable sensors are spreading worldwide, with a growth of *>*20% per year, expecting to increase to EUR 150 billion by 2028. Wearables have multiple advantages: they are user-friendly, relatively inexpensive, non-invasive, and available in several market segments [51]. Despite their advantages, multiple challenges related to the use of wearables in real practice are still unaddressed with regard to data collection, data processing, communications, and security. Regarding data acquisition, the quality, quantity, resolution, and other parameters of the gathered data are strictly dependent on the wearable device. Indeed, spatial resolution, temporal resolution, or data resolution of the device are the factors which may impact data quality and quantity [52]. Pre-processing also poses challenges in terms of application process unification and statistical outliers’ removal. Data processing often requires expert knowledge or the intervention of the wearable user [53].

To address these issues, artificial intelligence (AI) methods, both supervised and unsupervised, have recently been progressively applied [54]. AI techniques have been used in signal pre-processing and in the steps to denoise signals. For example, a combination of an active-contour-based loss and an adapted U-Net architecture has been applied to automatically detect regions of artifacts within plethysmography (PPG) signals [55]. AI methods can also be applied in the processing step to extract features and/or to classify the signals. For example, recurrent neural networks [56] or spiking neural networks [57] have been used to classify events in the ECG signals collected with wearables.

As a future direction, in a larger sample, we plan to develop and apply AI techniques in our studies in order to improve the quality of the collected signals and to analyze the data in order to, for example, predict the outcome of the training.

## 5. Conclusions

To summarize the knowledge generated by the present work, it was seen that a short, intensive sensory training devoted to wine tasters modulated the ANS activity toward a less sympathetically mediated response as soon as the odorous compounds became familiar. This is in line with previous retrievals on long, less intensive training courses, enabling a large-scale application of shorter formative courses in this domain, thus leading to money saving for academia and scientific societies, and challenging the dropout rates that might affect longer courses. Beyond the food and drink industry, if also confirmed in different populations (e.g., those with some clinically relevant conditions), this approach can be also translated into clinical practice and also possibly in the neuromarketing field. In the latter scenario, by adopting more pervasive devices (smartwatches, actigraphs, etc.), this approach is potentially capable of retrieving a larger amount of data that, when merged with artificial intelligence and machine learning approaches, might also be able to increase the knowledge of specific questions in this further particular scenario.

## Figures and Tables

**Table 1:** Model solutions used for the olfactory stimulation (Sessions T0 and T1).

Sample Code	Descriptor	Formulation
1	Raspberry	White wine * (100 mL); raspberry juice ** (80 mL)
2	Grapefruit	White wine * (100 mL); grapefruit juice ** (80 mL)
3	Orange	White wine * (100 mL); orange juice *** (80 mL)
4	Pineapple	White wine * (100 mL); pineapple juice *** (80 mL)
5	Fig	No diluted dried figs (10 g)
6	Asparagus	White wine * (100 mL); asparagus cooking water (60 mL) **
7	Peach	White wine * (100 mL); peach juice *** (80 mL)
8	Green pepper	Fresh green pepper (20 g) in 100 mL of white wine *
9	Mango	Dried mango (5 g) in 100 mL of white wine *
10	Rose	Distilled rose water (4 mL) + 100 mL of white wine *

* Tavernello; ** Esselunga Bio; and *** Skipper Zuegg.

**Table 2:** Difference in ECG features from T0 to T1. * *p* < 0.05 and ** *p* < 0.01.

Feature	T0	T1	*p*-Value
Baseline			
HR (bpm)	75.8 ± 6.9	69.8 ± 8.3	0.008 **
RMSSD (s)	0.036 ± 0.013	0.052 ± 0.030	0.016 *
NN50 (n.u.)	13.833 ± 8.533	18.909 ± 14.029	0.028 *
CSI (n.u.)	3.093 ± 0.754	2.359 ± 1.012	0.008 **
CVI (n.u.)	−2.790 ± 0.309	−2.716 ± 0.352	0.286
LF (s^2^/Hz)	−0.094 ± 0.828	−0.124 ± 0.782	0.091
HF (s^2^/Hz)	0.344 ± 0.232	0.690 ± 0.488	0.026 *
LF/HF (n.u.)	1.617 ± 2.147	0.655 ± 0.716	0.026 *
Task			
HR (bpm)	80.5 ± 9.0	72.6 ± 7.7	0.005 **
RMSSD (s)	0.027 ± 0.008	0.037 ± 0.016	0.022 *
NN50 (n.u.)	36.150 ± 39.568	62.960 ± 61.404	0.038 *
CSI (n.u.)	3.928 ± 0.902	3.145 ± 0.822	0.005 **
CVI (n.u.)	−2.912 ± 0.232	−2.789 ± 0.329	0.139
LF (s^2^/Hz)	0.172 ± 0.718	0.081 ± 0.834	0.169
HF (s^2^/Hz)	0.121 ± 0.098	0.415 ± 0.484	0.005 **
LF/HF (n.u.)	4.370 ± 3.647	2.166 ± 2.302	0.093
Recovery			
HR (bpm)	76.5 ± 7.8	71.5 ± 8.2	0.010 *
RMSSD (s)	0.027 ± 0.011	0.034 ± 0.013	0.026 *
NN50 (n.u.)	8.241 ± 5.763	11.633 ± 9.131	0.058
CSI (n.u.)	3.162 ± 0.854	2.913 ± 1.053	0.424
CVI (n.u.)	−3.029 ± 0.304	−2.874 ± 0.235	0.010 *
LF (s^2^/Hz)	−0.091 ± 0.867	−0.106 ± 0.890	0.722
HF (s^2^/Hz)	0.307 ± 0.182	0.553 ± 0.513	0.062
LF/HF (n.u.)	1.809 ± 1.588	1.407 ± 1.752	0.062

**Table 3:** Difference in GSR features from T0 to T1. * *p* < 0.05.

Feature	T0	T1	*p*-Value
Baseline			
Global (µS)	2.003 ± 1.683	0.994 ± 0.320	0.023 *
Tonic (µS)	1.792 ± 1.643	0.762 ± 0.420	0.019 *
Phasic (µS)	0.210 ± 0.226	0.232 ± 0.240	0.814
Task			
Global (µS)	3.099 ± 2.391	1.503 ± 0.772	0.012 *
Tonic (µS)	2.819 ± 2.278	1.164 ± 0.580	0.010 *
Phasic (µS)	0.280 ± 0.224	0.339 ± 0.339	0.530
Recovery			
Global (µS)	3.145 ± 2.394	1.528 ± 0.791	0.012 *
Tonic (µS)	2.941 ± 2.366	1.279 ± 0.763	0.015 *
Phasic (µS)	0.204 ± 0.185	0.248 ± 0.234	0.695

## Data Availability

The data presented in this study are available on request from the corresponding author. The data are not publicly available due to ethical reasons.
